# Generation and Applications of Induced Pluripotent Stem Cell-Derived Mesenchymal Stem Cells

**DOI:** 10.1155/2018/9601623

**Published:** 2018-07-31

**Authors:** Chengzhu Zhao, Makoto Ikeya

**Affiliations:** Department of Clinical Application, Center for iPS Cell Research and Application, Kyoto University, Kyoto 606-8507, Japan

## Abstract

Mesenchymal stem cells (MSCs) are adult stem cells with fibroblast-like morphology and isolated from the bone marrow via plastic adhesion. Their multipotency and immunoregulatory properties make MSCs possible therapeutic agents, and an increasing number of publications and clinical trials have highlighted their potential in regenerative medicine. However, the finite proliferative capacity of MSCs limits their scalability and global dissemination as a standardized therapeutic product. Furthermore, adult tissue provenance could constrain accessibility, impinge on cellular potency, and incur greater exposure to disease-causing pathogens based on the donor. These issues could be circumvented by the derivation of MSCs from pluripotent stem cells. In this paper, we review methods that induce and characterize MSCs derived from induced pluripotent stem cells (iPSCs) and introduce MSC applications to disease modeling, pathogenic mechanisms, and drug discovery. We also discuss the potential applications of MSCs in regenerative medicine including cell-based therapies and issues that should be overcome before iPSC-derived MSC therapy will be applied in the clinic.

## 1. Introduction

Mesenchymal stem cells (MSCs) are derived from the bone marrow, adipose tissue, or other connective tissues. Importantly, they have promise for tissue repair because of their expandability and multipotency. MSCs are able to differentiate into a broad spectrum of end-stage cell types such as osteoblasts, chondrocytes, myocytes, and adipocytes. Many reports have demonstrated that MSCs secrete a wide variety of bioactive molecules that exhibit immunoregulatory and microenvironment modulatory effects at the site of injury. These properties make MSCs as potential candidate for regenerative medicine.

However, MSCs derived from these cell sources have some limitations, including limited cell proliferative capacity and alterations in phenotype and differentiation potential during long-term culture [1]. Moreover, the quality of MSCs varies widely among donors [2–4]. Consequently, despite their potential, in some cases, MSCs have not translated well for the research and treatment of patients. Instead, an inexhaustible and safe source of MSCs is ideal.

MSCs induced from pluripotent stem cells (PSCs), such as embryonic stem cells (ESCs) and induced pluripotent stem cells (iPSCs), could provide such a source. Both possess properties of infinite growth and differentiation, making it possible to avoid long-term culture as MSCs. PSCs are also compatible with gene-directed enzyme prodrug therapy (GDEPT) or CRISPR/Cas9 and related technologies [5, 6] for gene editing. Moreover, iPSCs can be obtained with minimally invasive procedures and avoid the key ethical controversy surrounding ESCs regarding embryo use [7–9]. They also minimize immunologic problems when autologous or HLA-matched iPSC lines are used [10]. These characteristics enable the study of MSC-related diseases, drug screening using patient cells, and the transplantation of large quantities of cells for cell therapy opening a new avenue for translational medicine.

However, several issues must first be answered before iPSC-derived MSCs (iMSCs) fulfill this potential. For example, the key signals and optimum protocols for effective differentiation and criteria for the evaluation of the clinical quality and safety of iMSCs are still to be determined. In this article, we describe recent research pertaining to the differentiation methods of MSCs from iPSCs, the application of iMSCs to disease modeling and drug screening, animal experiments using iMSC-based cells for preclinical study, and challenges that should be overcome before clinical application.

## 2. Derivation of MSCs from iPSCs

The past ten years have seen a great rise in efforts to generate MSCs from PSCs. Multiple protocols exist to derive cells with the features that characterize MSCs. The original strategy to generate MSCs from PSCs involved depriving the culture medium of pluripotent signals, which resulted in spontaneous differentiation to MSCs that mechanically separated from PSCs [11–13]. Although these cells exhibit MSC morphology and express MSC surface markers, the differentiation induction was inefficient. Later, researchers showed that it is possible to obtain MSCs by using a medium supplemented with bFGF [14–16]. These cells are highly similar to MSCs with regard to morphology and expression of markers. However, they have diminished differentiation potential, particularly toward adipogenic lineage [15].

**Figure 1:**
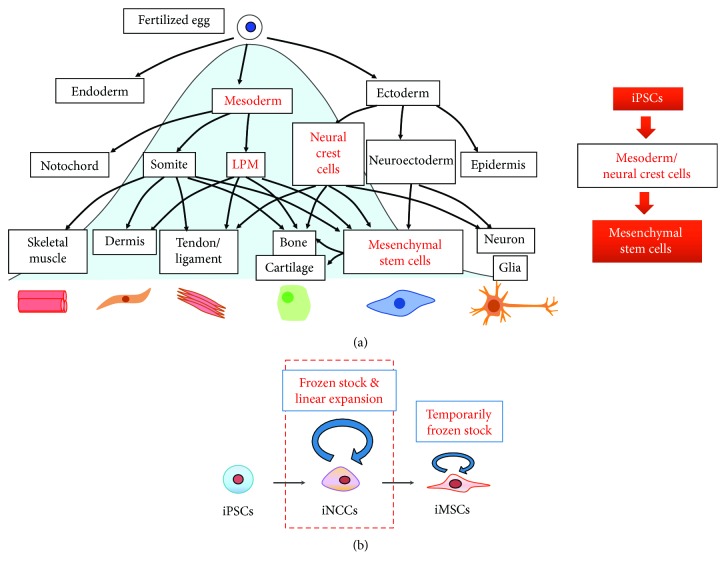
Derivation of MSCs from iPSCs. (a) Inducing iMSCs from iPSCs by mimicking embryonic development. During embryonic development, MSCs arise from two major sources: mesoderm and neural crest cells (left). Protocols for the induction include specific signals and morphogens that drives the iPSCs to mesoderm/NCC formation and then generate iMSCs (right). LPM: lateral plate mesoderm. (b) Protocols that induce iMSCs from iPSCs through iNCCs result in a homogeneous mesenchymal cell population without contamination of other cellular phenotypes; therefore, when differentiation is complete, no remnants of undifferentiated cells are found. Frozen stocks can be made to use cells of the same quality in order to evaluate reproducibility.

The regulation of signaling that mimics embryonic development is considered necessary to ensure MSCs derived from PSCs exhibit predictable properties and functions [17]. Although the developmental origins of MSCs are not fully understood, somatic lateral plate mesoderm is considered to be the major source of MSCs (LPM in Figure 1(a)) because it gives rise to the majority of the body's adipose and skeletal tissues [18, 19]. Based on better understanding of the critical signals for mesoderm, several reports have shown the induction of mesoderm and then MSCs using known morphogens. For example, Mahmood et al. inhibited TGF*β* signaling with the use of SB-431542 during PSC differentiation in embryoid body [18]. Sánchez et al. used a similar strategy but this time in cells that grew only in two dimensions (2D) [19]. These protocols were demonstrated to be effective in differentiating PSCs into MSCs, as the derived cells expressed mesenchymal surface markers and possessed multipotency and immune regulation activity.

Neural crest was identified as another developmental origin of MSCs [20, 21]. Simple and efficient methods to generate MSCs through neural crest cell lineage from iPSCs have been established by several groups [22–28]. The activation of canonical Wnt signaling and the prevention of TGF*β* signaling are common approaches used to obtain a highly enriched induced neural crest cell (iNCC) population. To establish an ideal method with respect to clinical application, feeder-free and serum-free culture should be performed using coating material and chemically defined medium (CDM). Some reported protocols are suitable for these demands [22, 25, 27]. For example, Menendez et al. employed a two-step approach that first dissociated iPSCs into single cells, then cultured them for two weeks in the CDM to achieve feeder-free, serum-free condition, and finally supplemented the culture with Wnt signaling activator and Activin/Nodal/TGF*β* signaling inhibitor [25]. Mica et al. cultured PSCs in MEF-conditioned hESC media, then substituted the medium with knockout serum replacement- (KSR-) based medium, and finally replaced KSR gradually with increasing amounts of N2 medium. Next, BMP signaling and Activin/Nodal/TGF*β* signaling inhibitors were included and then replaced with a Wnt signaling activator [27]. Our group has developed a strong and efficient iMSC generating method using CDM containing TGF*β* and GSK3*β* signaling inhibitors with minimal growth factors [22]. This protocol generated iNCCs (70–80%) independent of the human PSC generation method (viral-integrated or plasmid-episomal). iNCCs can be expanded a long time under conditions of bFGF and EGF supplementation and TGF*β* inhibition, and our protocol could generate a homogeneous, completely differentiated population of MSCs. Moreover, frozen stocks of both iNCCs and iMSCs can be made (Figure 1(b)) providing greater convenience for future clinical use.

### 2.1. Characterization of iMSCs

iMSCs exhibit plastic adherence, express MSC surface markers, and can differentiate into osteoblasts, adipocytes, and chondroblasts. These properties satisfy the minimal criteria of human MSCs proposed by the International Society of Cellular Therapy [29, 30]. Genome-wide expression profiles of iMSCs were compared to well-defined MSC types, such as adult bone marrow-derived MSCs (BM-MSCs), and significant overlap of both types in gene expressions has been shown [22, 31–34]. Meanwhile, iMSCs maintained gene expressions and DNA methylation profiles in accordance with the initial donor, except for tissue-specific and age-related DNA methylation profiles [35]. The marker expression pattern in iMSCs was distinct from iPSCs. Namely, the expression of CD-326, Tra-1-60, SSEA-4, and E-cadherin were diminished [29]. These features should be considered before the application of iMSCs to animal experiments and preclinical trials.

When considering the main source for the derivation of iPSCs, it should be noted that human dermal fibroblasts (HDFs) exhibit phenotypic similarity to MSCs. For example, the same surface phenotype (CD73^+^, CD90^+^, and CD105^+^ cell level), immunosuppressive ability [36–39], and even osteo/chondro/adipo differentiation ability [40] between HDFs and MSCs were reported. These properties may interfere with cell identification during iMSC application. Highly similar gene and microRNA expression patterns of HDFs and MSCs were reported, suggesting overlapping phenotypic and functional properties [41].

Because some clinical iPSC lines were derived from HDFs, ways to separate HDFs from HDF-derived iMSCs are necessary to apply these lines. However, at present, there is no reliable marker for HDFs differentiated from HDF-derived iMSCs, but there do exist a number of candidates. TM4SF1 is a surface protein that is highly expressed in various MSC sources but not in fibroblasts [42]. A genome-wide oligonucleotide microarray analysis of HDFs and MSCs might also be beneficial. Additionally, a number of transmembrane protein, tumor, and metastasis-related genes have been found to be upregulated 10-fold in MSCs compared with HDFs, while HDFs show significantly higher surface antibody CD10 levels. Culture conditions that induce MSCs to differentiate into osteocytes, adipocytes, and chondrocytes do not induce HDFs to differentiate into any of these lineages [41]. Together, these properties could be used to identify iMSCs.

### 2.2. iMSC-Based Disease Modeling and Drug Discovery

Clarifying the pathological mechanisms underlying human diseases is important for the discovery of novel therapeutic strategies for genetic diseases. Because of limitations in patient tissue and the lack of appropriate animal models, research on these genetic disorders remains challenging. Cellular disease models using patient-specific iPSCs provide new understanding of these diseases. iMSCs differentiated from patient iPSCs not only act as ideal tools for pathologic research but also provide platforms for drug screening and toxicity testing.

Deyle et al. [43] reported an iPSC model for osteogenesis imperfecta (OI), a genetic bone disorder caused by a dominant mutation in type 1 collagen genes. They generated transplantable patient-specific iMSCs differentiated from OI patient iPSCs in which the collagen mutation was removed. These cells could act as bone-forming cells for the purpose of treating defects in the skeletal tissue of OI patients.

Liu et al. modeled Fanconi anaemia (FA), a rare disease caused by an impaired response to DNA damage, using patient-derived iMSCs. FA-iMSCs showed impairment in maintenance and proliferation, similar to MSCs from *Fancg*-deficient mice [44].

Zhang et al. prepared iMSCs from patients with Hutchinson-Gilford progeria syndrome (HGPS) to study the pathology. HGPS is a segmental premature aging disease that affects mesenchymal lineages and is caused by progerin, a truncated and farnesylated form of Lamin A. HGFP-iMSCs exhibit abnormalities including increased nuclear dysmorphology, DNA damage, and an accumulation of calponin-staining inclusion bodies, which are all properties consistent with fibroblasts isolated from HGPS patients. Compromised viability of HGFP-iMSCs under stress was observed *in vitro* and *in vivo*, especially to hypoxia. Reducing progerin levels by shRNA restored the ability of HGPS-iMSCs to resist hypoxia. Researchers have suggested that progerin toxicity makes HGPS-iMSCs overly sensitive to their hypoxic microenvironment, leading to exhaustion of the MSC pool caused by replacing lost mesenchymal tissue [45]. Cicero et al. performed a high-throughput screening of 2800 small molecules that could inhibit the differentiation of HGPS-iMSCs towards osteogenic lineage by monitoring alkaline phosphatase activity (ALP). They identified seven compounds that significantly decreased premature osteogenic differentiation, four of which decreased progerin expression [46].

Fibrodysplasia ossificans progressiva (FOP) is a rare genetic disease that is characterized by progressive heterotopic ossification (HO) in connective tissues. It is caused by mutations in the ACVR1 gene, which encodes Activin-A receptor type 1, an important protein in the bone morphogenetic protein (BMP) pathway. Our group [47, 48] generated FOP patient-derived iPSC clones and gene-corrected (rescued) iPSC clones (resFOP-iPSC). iMSCs were induced from both iPSC clones through neural crest cell lineage. FOP-iMSCs exhibited augmented chondrogenic ability and enhanced activity of the SMAD1/5/8 pathway compared to resFOP-iMSCs, successfully recapitulating the disease phenotype. Using these cells, we screened TGF*β* superfamily ligands that could specifically activate BMP signaling through FOP-ACVR1 by a luciferase reporter (BRE-Luc) assay and found a new FOP mechanism. Activin-A, a TGF*β* signal transducer, evokes BMP signaling activation via FOP-ACVR1. To develop an *in vivo* evaluation system, FOP- and resFOP-iMSCs were transplanted together with Activin-A-expressing C3H10T1/2 cells into the skeletal muscle of immunodeficient mice. HO was developed at the transplanted site after six weeks, suggesting Activin-A induces extraskeletal bone formation in FOP [49].

To reveal the molecular mechanisms of the enhanced chondrogenesis evoked by Activin-A and to discover potential therapeutic targets, a high-throughput screening (HTS) system was established using FOP-iMSCs. From the screening of nearly 7000 small-molecule compounds, the mTOR signaling pathway was identified as important in the excessive chondrogenesis seen in FOP-iMSCs. Rapamycin, a commonly used mTOR inhibitor and commercially available drug, repressed HO in FOP-iMSC-transplanted mouse extensively. According to a DNA microarray assay, ENPP2 (also known as autotaxin) was indicated to act upstream of mTOR signaling, upregulating the chondrogenesis activity of FOP-iMSCs in response to Activin-A [50].

**Figure 2:**
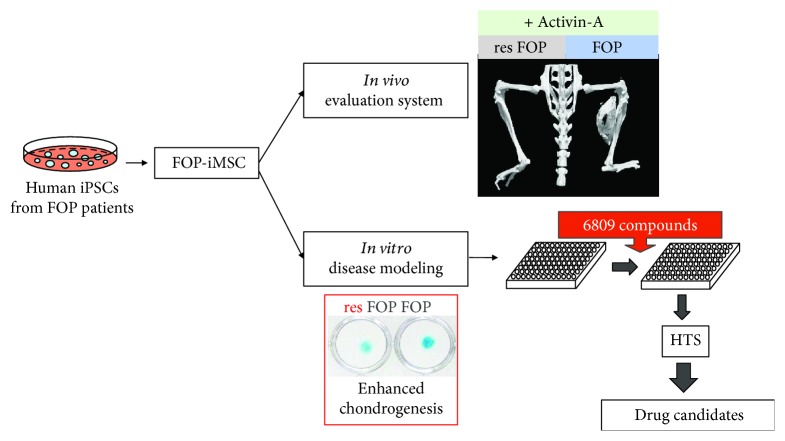
iMSC-based disease modeling and drug discovery of FOP. iMSCs generated from FOP patient-derived iPSC clones (FOP-iMSC) and gene-corrected (rescued) iPSC clones (resFOP-iPSC) could be applied to *in vitro* disease modeling, drug screening, and *in vivo* drug efficacy evaluation.

The FOP study shows that iMSC-based hereditary disease models and drug discovery platforms can reliably reproduce disease phenotypes and offer tremendous advantage for exploring drug candidates and critical disease mechanisms, accelerating the development of novel therapies (Figure 2).

### 2.3. iMSC-Based Experimental Therapies and Challenges

As the origin of iMSC derivation, iPSCs can be obtained from any adult tissue source and used to generate an abundance of iMSCs at low passage [51]. Furthermore, the abilities of iMSCs to differentiate into multiple tissues, produce a broad variety of cytokines and paracrine factors, regulate immune response, secrete exosomes, and exhibit mitochondrial transfer function suggest they could be potential cell sources for therapeutic purposes.

A number of animal studies using iMSCs have shown significant benefits on tissue regeneration and repair. iMSCs promote periodontal regeneration and new mineralized tissue formation when implanted into rat periodontal defect models [29]. The transplantation of iMSC-induced osteoblast into the calvarial defects of mice was found to support bone formation at the defect site [46]. iMSCs also exert stem cell factor-dependent recovery of cigarette smoke-induced apoptosis/proliferation imbalance in airway cells [52]. After being implanted into an ischemic site in mouse hindlimb, iMSCs significantly attenuated the physiological status to a degree superior than adult BM-MSCs [53]. The repair ability of iMSCs in myocardial infarction, cigarette smoke-induced cardiac remodeling, and dysfunction mouse model has also been observed [54, 55]. Similar to the ischemia in hindlimb, the cardiac wound repairing and proangiogenic potency of iMSCs were superior to those of BM-MSCs and umbilical cord blood-derived MSCs [56]. The greater therapeutic potential of iMSCs may be due to their superior survival and engraftment (for more than 5 weeks) after transplantation [52].

In addition, iMSCs are safe and efficient as immune modulators in both inflammation and autoimmunity diseases, presumably due to their ability to suppress abnormal immune response [57–59]. An *in vitro* study indicated that iMSCs remarkably impair the proliferation and cytolytic function of NK cells. Again, this effect by iMSCs was stronger than that of BM-MSCs, suggesting iMSCs could be a useful therapeutic choice to inhibit allograft rejection [38]. After transplantation into streptozotocin-diabetic mice, iMSCs ameliorated diabetic polyneuropathy (DNP) [60].

Exosomes assembled from iMSCs exhibit therapeutic potential in several animal disease models [56, 61]. Recent studies report that tissue repairing ability of iMSC via mitochondrial transfer mechanism exhibit superior effect on the mouse model of anthracycline-induced cardiomyopathy and cigarette smoke-induced lung damage compared with BM-MSCs [62, 63].

There are a large number of clinical trials using MSCs in various diseases, including cardiovascular diseases, hepatic diseases, neurological disorders, and autoimmune diseases (http://ClinicalTrials.gov). However, before iMSCs can serve as an alternative source of MSCs in the clinic, several obstacles must be overcome [64]. Because they are derived from iPSCs, iMSCs need to be carefully tested for alterations in oncologic genes [65]. The classic method to induce iPSCs involved the use of a retrovirus to overexpress c-Myc, a protooncogene that increases reprogramming efficiency by inhibiting the tumor repressor gene p53, which can increase the probability of tumor formation. Safe and effective factors along with nonviral gene delivery systems are expected to enhance the safety profile of iMSCs for clinical application [66–70]. Furthermore, the purity and quality of the iMSCs must be considered. Sorting iMSCs by the positive expression of MSC markers and the negative expression of pluripotency markers and testing the potential of oncogenesis on animal models should be performed before advancing iMSCs to the clinic [71].

## 3. Conclusion and Future Perspectives

iMSCs are a potentially abundant source of MSCs for disease modeling, drug discovery, and regenerative medicine [27]. To further advance iMSC-based therapeutic applicability and minimize the risk of immunoreaction after administration, it is necessary to optimize iPSC and iMSC production protocols. Removing oncogenic or other unstable factors and employing xeno-free culture condition at the reprogramming and differentiation induction step are necessary. The combination of new approaches into iMSC platforms, such as 3D organoid and gene editing technologies, might render iMSCs more valuable for further clinical application.
